# Histologically Overt Stromal Response and the Risk of Progression after Radical Prostatectomy for Prostate Cancer

**DOI:** 10.3390/cancers16101871

**Published:** 2024-05-14

**Authors:** Mutlay Sayan, Yetkin Tuac, Samet Kucukcolak, Mary D. Rowan, Grace K. Pratt, Cagdas Aktan, Elza Tjio, Dilara Akbulut, Shalini Moningi, Jonathan E. Leeman, Peter F. Orio, Paul L. Nguyen, Anthony V. D’Amico, Mahmut Akgul

**Affiliations:** 1 Department of Radiation Oncology, Brigham and Women’s Hospital and Dana Farber Cancer Institute, Harvard Medical School, Boston, MA 02115, USA; 2 Department of Statistics, Ankara University, 06100 Ankara, Türkiye; 3 Department of Pathology and Laboratory Medicine, Rutgers University, New Brunswick, NJ 07102, USA; 4 Department of Medical Biology, Faculty of Medicine, Bandirma Onyedi Eylul University, 10250 Balikesir, Türkiye; 5 Histopathology Department, Harrogate District Hospital, Harrogate HG2 7SX, UK; 6 Laboratory of Pathology, Center for Cancer Research, National Institutes of Health, Bethesda, MD 20892, USA; 7 Department of Pathology and Laboratory Medicine, Albany Medical Center, Albany, NY 12208, USA

**Keywords:** prostate cancer, HOST-response, radical prostatectomy, adjuvant therapy, progression-free survival

## Abstract

**Simple Summary:**

Prostate cancer is a commonly diagnosed cancer in men with varying outcomes following treatment. This study focuses on a specific feature observed in some prostate cancers called a histologically overt stromal response (HOST-response), which involves changes in the tissue surrounding the cancer cells. Our research used data from patients who had undergone surgery for prostate cancer to explore how this stromal response impacted their recovery without the cancer returning. We found that the presence of a HOST-response is linked to a higher chance of the cancer coming back sooner. These findings suggest that monitoring for this feature could help identify patients who might benefit from additional treatments after surgery, which could potentially improve their outcomes. The results of this study could guide future clinical trials aiming to refine treatment strategies for prostate cancer patients exhibiting a HOST-response.

**Abstract:**

Purpose: Given the variable clinical course of prostate cancer and the limitations of current prognostic factors, this study was conducted to investigate the impact of a histologically overt stromal response (HOST-response) to prostate cancer on clinical outcomes after radical prostatectomy. Methods: This retrospective analysis utilized The Cancer Genome Atlas (TCGA) to evaluate data from individuals with a confirmed diagnosis of prostate cancer who underwent radical prostatectomy and had available pathology slides. These slides were assessed for the presence of a HOST-response, similar to desmoplasia. The primary endpoint was progression-free survival (PFS). A multivariable competing risk regression analysis was used to assess whether a significant association existed between HOST-response and PFS, adjusting for known prostate cancer prognostic factors. Results: Among the 348 patients analyzed, 166 (47.70%) demonstrated a HOST-response. After a median follow-up of 37.87 months (IQR: 21.20, 65.50), the presence of a HOST-response was significantly associated with a shorter PFS (SDHR, 2.10; 95% CI, 1.26 to 3.50; *p* = 0.004), after adjusting for covariates. Conclusions: HOST-response in prostate cancer patients treated with radical prostatectomy is significantly associated with reduced PFS, suggesting a potential benefit from adjuvant therapy and highlighting the need for further investigation in a prospective randomized clinical trial.

## 1. Introduction

Prostate cancer is a morphologically heterogenous disease characterized by a variable clinical course, which emphasizes the importance of precise prognostication to avoid both over- and undertreatment [1,2,3,4]. Current methods, including the evaluation of the Gleason score and serum prostate-specific antigen (PSA) levels, are standardly employed to predict disease outcomes. However, these prognostic factors sometimes fail to provide the precision required for optimal clinical decision-making, leading to potential gaps in treatment strategies [5,6,7]. This deficiency underscores an urgent demand for the development and integration of more reliable and specific diagnostic indicators that can better guide the therapeutic course and management of prostate cancer patients.

In this context, the stromal microenvironment has emerged as a clinically significant histological pattern, offering new insights into the mechanisms of cancer progression [8,9,10,11]. Characterized by significant alterations driven by carcinoma-associated fibroblasts (CAFs), the reactive stroma plays a pivotal role in various aspects of cancer progression, including tumor growth, angiogenesis, and the malignant transformation of epithelial cells [12,13]. Prostate cancer with intra- and inter-stromal fibrous tissue growth, similar to desmoplasia, or histologically overt stromal response (HOST-response), is based on the identification of a desmoplastic stroma composed of myofibroblasts instead of native smooth muscle cells with a stroma/epithelium ratio ≥ 1 and is frequently associated with a higher Gleason Score. The “Stromogenic prostatic carcinoma” term has also been used to describe tumors showing a HOST-response. The presence of a reactive stroma alters the tumor glands, too, with a characteristic angulated and distorted appearance. Besides the histopathologic difference, differential gene expression has also been identified for the reactive stroma, compared to the native smooth muscle of the prostate [14]. Consequently, the term “stromogenic prostatic carcinoma” has been suggested as a specific histological pattern, highlighting the reactive stroma’s role not merely as a part of the tumor microenvironment but as a critical determinant of the cancer’s prognosis [8,15,16,17].

In prostate cancer, a HOST-response has been identified as a significant factor affecting patient outcomes, notably linked to both biochemical recurrence and prostate cancer-specific mortality [11,18,19]. Recognizing its potential to refine prognostic accuracy and inform treatment strategies, a more comprehensive understanding of a HOST-response’s role in prostate cancer is imperative. In this study, we investigate the impact of a HOST-response on progression-free survival while adjusting for known prostate cancer prognostic factors.

## 2. Materials and Methods

### 2.1. Patient Characteristics

In this retrospective investigation, we analyzed individual patient records from The Cancer Genome Atlas (TCGA), a comprehensive database collecting diverse cancer-related data. Eligibility for inclusion in this study required patients to have a verified diagnosis of prostate cancer, to have completed radical prostatectomy, and to have digital slides stained with hematoxylin and eosin (HE) available that clearly delineated the tumor regions for accurate assessment.

The baseline demographic and clinical information for each patient, which included the age at diagnosis, the pathological stage of the tumor, the status of surgical margins, the Gleason score, and the levels of PSA prior to treatment, were collected. These specific variables were selected due to their proven significance in predicting the prognosis and determining the therapeutic approaches for prostate cancer. The period of follow-up for this study extended from the date of the radical prostatectomy until the last recorded follow-up or death of the patient, whichever occurred first.

**Figure 1:**
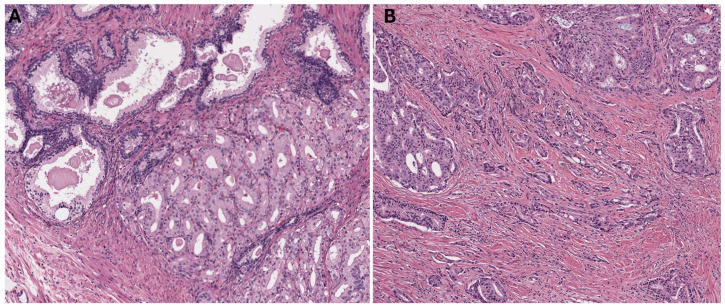
Representative cases of prostate cancer without histologically overt stromal response (HOST-response) at 100× magnification (**A**) and with HOST-response at 40× magnification (**B**).

An evaluation of the radical prostatectomy specimens was conducted rigorously by a team of three pathologists specialized in genitourinary disorders (identified as MA, ET, and SK). Their primary task was to determine the occurrences of a HOST-response in radical prostatectomy specimens. To achieve this, the pathologists applied a series of predefined histological standards designed to maintain the uniformity and precision of the evaluations across all samples. HOST-response refers to peri-glandular stromal changes causing desmoplasia-like changes in the stroma as well as architectural changes in the prostate cancer glands, most commonly angulation as well as occasionally retraction artifacts [8,9]. These changes may sometimes cause confusion in prostate cancer grading assessment. Despite previous reports on grading the prostate cancer HOST-response, the reproducibility of finding a HOST-response is highest in grade 3 stromogenic changes [8]. Therefore, our definition of a HOST-response refers to the changes previously described as grade 3. Tumors with >50% of reactive stroma area relative to the total tumor area were given stromal grade 3 (Figure 1) [8].

### 2.2. Statistical Methods

#### 2.2.1. Comparison of the Distribution of Baseline Clinical Characteristics Stratified by HOST-Response

A comprehensive statistical analysis plan was carefully designed and strictly followed during the execution of this study to ensure robustness and accuracy in the results. Descriptive statistics were used to systematically analyze the clinical and treatment characteristics of the 348 patients. These patients were stratified based on the presence or absence of a HOST-response, which guided subsequent statistical assessments. Age and baseline PSA (ng/mL) data were collected as continuous covariates. Median and interquartile range (IQR) values were calculated to identify their descriptive statistics. The patients were categorized by prostatectomy tumor stage (“T2” and “T3a or higher”), prostatectomy Gleason score (“7 or less” and “8–10”); prostatectomy margin status (“Negative” and “Positive”); prostatectomy nodal status with the categories of (“Negative (N0)” and “Positive (N1)”); and HOST-response (presence or absence). The counts and the percentages of these categorical variables are calculated to obtain the descriptive statistic results. For categorical data, a Pearson’s chi-squared test was utilized to evaluate the statistical significance of differences between groups. Frequencies and percentages for each category within these variables were calculated and presented in a comparative table (Table 1) to provide a clear visual differentiation of their distributions. Continuous variables, including age and PSA levels, were analyzed using the Wilcoxon two-sample test [20]. This non-parametric method was chosen due to its effectiveness with data distributions that do not assume normality. These variables were reported using medians and IQRs, allowing for a robust comparison between the patient groups. Additionally, the study involved comparing the distribution of follow-up times, an important factor in longitudinal studies assessing treatment outcomes and survival rates. The Kaplan–Meier method was applied to generate survival curves for the patient groups. These curves were then analyzed using a log-rank test to determine *p*-values, offering a statistical measure of the differences in survival distributions across the studied cohorts. The distribution of the follow-up times was assessed using the reverse Kaplan–Meier method [21]. Finally, univariate and multivariate competing risk regression analysis using the Fine and Gray regression [22] was performed to evaluate whether a significant association existed between the presence of a HOST-response and PFS. Fine and Gray regression was used to model PFS on the risk of biochemical recurrence, with death as a competing risk.

#### 2.2.2. Covariate Subdistribution Hazard Ratios for Progression-Free Survival

The primary outcome of this study was progression-free survival (PFS), which was defined as the duration from the completion of radical prostatectomy to cancer progression or death from any reason, whichever came first [23,24,25]. To evaluate the impact of HOST-response on PFS, both univariable and multivariable competing risk regression analyses were used, following the methodological framework established by Fine and Gray. The reference point, or Time 0, for these analyses was set as the date on which the radical prostatectomy was performed. The model incorporated adjustments for several critical covariates, including age, pre radical prostatectomy PSA, tumor stage at the time of radical prostatectomy, Gleason score, and margin status. Subdistribution hazard ratios (SDHRs) with 95% confidence intervals (CIs) and associated *p*-values for each clinical covariate were reported.

#### 2.2.3. Adjusted Estimates of Progression-Free Survival

For the purpose of illustration, the covariate-adjusted estimates of PFS following radical prostatectomy, stratified by the presence or absence of a HOST-response, were determined through the Kaplan–Meier method [26]. The calculation of these PFS estimates included adjustments for several key covariates such as age at the time of radical prostatectomy, PSA levels, the stage of the tumor at the time of radical prostatectomy, Gleason score, and the status of surgical margins. Confidence intervals at the 95% level were also computed to provide a range of expected outcomes within the context of these variables.

To ascertain the statistical significance of differences observed in the Kaplan–Meier survival plots between groups, the log-rank test was utilized. This test helped us to evaluate whether the variations in survival distributions could be attributed to a HOST-response. A significance threshold was established at *p* < 0.05, enabling the determination of statistically significant findings.

The entirety of these statistical evaluations were conducted using the R software (version 4.2.3), ensuring rigorous data analysis and the integrity of the results presented.

## 3. Results

### 3.1. Description and Comparison of the Distribution of Baseline Clinical Characteristics Stratified by HOST-Response

Among the 348 patients analyzed, 166 (47.70%) showed a HOST-response. Detailed characteristics of these patients, categorized based on whether they have a HOST-response, are systematically presented in Table 1. In the comparative analysis of patients grouped by the presence or absence of a HOST-response, notable differences emerged in several clinical measures. Specifically, the duration of follow-up for patients with a HOST-response was significantly longer (*p* = 0.004), with a median of 45.47 months (IQR: 24.13, 68.53) compared to a median of 32.63 months for those without a HOST-response (IQR: 17.47, 60.50). Additionally, baseline PSA levels were higher in the HOST-response group compared to those without a HOST-response [8.15 ng/mL (IQR: 5.4, 13.5) versus 7.20 ng/mL (IQR: 4.7, 10.4), *p* = 0.010]. The patients with a HOST-response had a significantly higher Gleason score when compared to patients without a HOST-response (45% versus 33%, *p* = 0.026). Furthermore, a higher proportion of patients with a HOST-response were diagnosed with advanced prostate cancer, specifically stages T3a or higher, affecting 68% of such patients versus 56% of those without a HOST-response (*p* = 0.024). However, the incidence of positive surgical margins post-prostatectomy showed no significant difference between the groups, with 28% in the HOST-response group and 37% in the non-HOST-response group (*p* = 0.089).

### 3.2. Univariable and Multivariable Hazard Ratios for Progression-Free Survival

After a median follow-up of 37.87 months (IQR: 21.20, 65.50), 76 (21.84%) men had biochemical recurrence, and 8 (2.29%) men died. Data presented in Table 2 reveal that the presence of a HOST-response was associated with a significantly lower PFS (SDHR, 2.10; 95% CI, 1.26 to 3.50; *p* = 0.004) after adjusting for known covariates.

### 3.3. Adjusted Estimates of Progression-Free Survival

**Figure 2:**
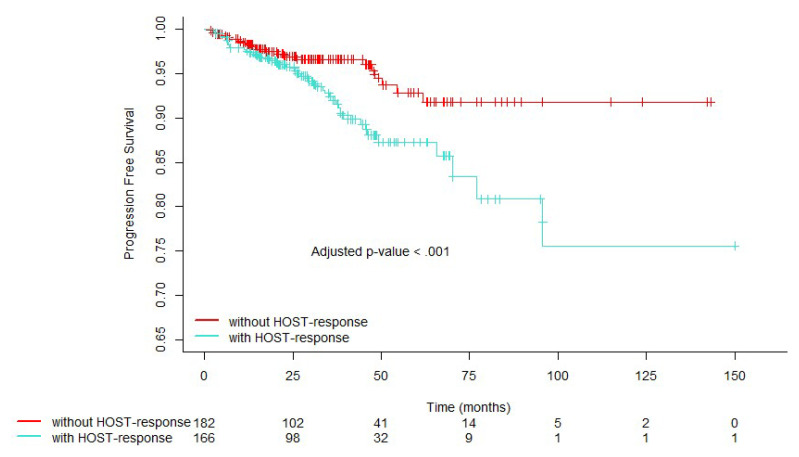
Covariate-adjusted estimates of progression-free survival stratified by the presence of a histologically overt stromal response (HOST-response).

Figure 2 illustrates the adjusted PFS estimates stratified by the presence or absence of a HOST-response. Estimates of the adjusted PFS were significantly lower (*p* < 0.001) in men with a HOST-response compared to those without it. The adjusted 10-year estimate of PFS for men with a HOST-response was 75.60% (95% CI, 57.70% to 99.00%) versus 91.81% (95% CI, 85.25% to 98.88%) for those without a HOST-response. The median overall survival (OS) was 31.65 months (IQR: 18.02, 52.44) and median PFS was 22.06 months (IQR: 1.94, 40.57).

## 4. Discussion

In this study, we found that in patients with prostate cancer treated with radical prostatectomy, the presence of a HOST-response in surgical pathology specimens was associated with a reduced PFS after adjusting for known prognostic factors of prostate cancer. The clinical implications of these findings are substantial, as they help to identify a specific group of patients, those with particularly adverse pathological features, who are at a higher risk of rapid disease progression.

In prostate cancer, the HOST-response significantly influences disease progression. This response encompasses alterations in the tumor microenvironment, primarily characterized by an increase in CAFs and a shift in the composition of the extracellular matrix (ECM) [9,12,13,27]. CAFs actively secrete a range of cytokines, growth factors, and enzymes such as TGF-β, VEGF, and matrix metalloproteinases (MMPs), which collectively promote tumor growth, angiogenesis, and metastatic spread [9,28]. Additionally, the reactive stroma can alter the immune landscape by creating an immunosuppressive microenvironment that aids tumor cells in evading immune surveillance [29,30]. The dense ECM serves not only as a physical barrier impeding therapeutic agent delivery but also fosters a protective niche for cancer cells against conventional therapies. These stromal-mediated mechanisms are pivotal in driving the aggressive behavior of the cancer, suggesting that a HOST-response is not merely a bystander effect but a potent facilitator of cancer progression. Therefore, understanding and targeting these stromal interactions presents a promising avenue for developing novel therapeutic strategies that address both the cancer cells and their supportive microenvironment.

Understanding that the HOST-response can be a marker of more aggressive disease provides a critical opportunity for clinical intervention. Specifically, this insight allows for the strategic selection of patients who could benefit most from additional therapeutic measures post-surgery. These patients are ideal candidates for inclusion in prospective, randomized clinical trials that are designed to investigate the effectiveness of adjuvant treatment following radical prostatectomy. Such trials could explore various adjuvant therapies that may mitigate the risk of recurrence and progression in this patient group. By tailoring clinical approaches to the unique pathology demonstrated by these patients, it is possible to enhance treatment outcomes significantly. This strategy not only has the potential to improve survival rates but also to refine the standard of care for prostate cancer patients with a HOST-response, leading to more personalized and effective treatment plans.

This analysis raises several important considerations that warrant further exploration. Firstly, the nature of this investigation as a retrospective study introduces inherent confounding factors that a non-randomized design may not fully address. As such, the conclusions drawn here primarily serve to generate hypotheses that need to be verified through more controlled experimental setups, such as a prospective cohort study. Additionally, to robustly validate our findings, the inclusion of an independent external cohort is essential. This approach would not only enhance the credibility of the results but would also help in assessing the reproducibility and generalizability of our findings across different populations.

Secondly, the histological assessments were based on annotations from one or two virtual slides per case. Given that the pathology review committee did not have access to complete prostatectomy specimens, there is a possibility that some cases were misclassified regarding the presence of a HOST-response. Specifically, a HOST-response might be present in portions of the specimen that were not included in the slides reviewed. This limitation highlights the need for comprehensive sample analysis to avoid potential biases in the evaluation of HOST-response presence. Despite this constraint, a notable association between the presence of a HOST-response and reduced PFS was identified, which remained significant even after adjusting for well-known prognostic factors of prostate cancer. This suggests that a HOST-response may indeed be an important marker of disease progression in prostate cancer.

Thirdly, it is important to mention that patients exhibiting a HOST-response frequently presented with higher Gleason scores and were more likely to have advanced-stage disease (T3a or higher). However, these variables were carefully adjusted for in the multivariable analysis to isolate the effect of a HOST-response from these confounding clinical factors. This adjustment ensures that the impact of a HOST-response on PFS is evaluated with an understanding of its independent contribution to the risk profile of the patients studied.

Lastly, the role of biochemical failure as the primary determinant of PFS in this study aligns with findings from previous research, which also noted a strong link between a HOST-response and biochemical failure [11,18]. Ayala et al. conducted extensive analysis on the role of the reactive stroma in prostate cancer, showing that the volume of the reactive stroma and expression of certain stromal markers were significant predictors of biochemical-free recurrence [11]. Utilizing tissue microarrays from 847 patients, it was found that specific alterations in tumor stromal components, particularly a decrease in desmin and smooth muscle α-actin, were independently predictive of recurrence-free survival [11]. These findings suggest that reactive stroma is not merely a passive component but actively influences the progression of prostate cancer. This study demonstrated that higher volumes of reactive stroma were associated with poorer outcomes, highlighting the prognostic significance of stromal elements in cancer pathology. In a continuation of their investigations on stromal influence, Ayala et al. aimed to correlate the extent of the reactive stroma with prostate cancer-specific mortality in a subsequent publication [18]. By quantifying the area of a HOST-response, also known stromogenic carcinoma, in prostatectomy specimens, they established that larger areas of the reactive stroma were directly associated with a higher risk of cancer-specific death. The study’s methodology involved reviewing entire prostatectomy samples to measure the percentage of HOST-responses and employing statistical tools like Spearman, Kaplan–Meier, and Cox analyses to validate their findings. The results reinforced the concept that a reactive stroma serves as a critical prognostic marker, not only for recurrence but also for mortality in prostate cancer patients. More recently, in a comprehensive population-based study, Sæter et al. explored the prognostic value of the reactive stroma, graded on needle biopsies, in predicting prostate cancer-specific mortality among patients without metastasis at diagnosis [19]. Analyzing 318 patients diagnosed between 1991 and 1999, the study found that higher grades of reactive stroma were strongly linked to an increased risk of prostate cancer-specific mortality over a median follow-up of 110 months. This association persisted even after adjusting for traditional prognostic factors such as PSA levels, clinical stage, Gleason score, and mode of treatment in a multivariate Cox regression analysis. The findings emphasized the independent prognostic value of a reactive stroma in needle biopsies, suggesting that HOST-response could enhance the predictive accuracy of existing prognostic models and aid in treatment stratification for prostate cancer patients.

These studies collectively highlight the clinical importance of evaluating reactive stroma in prostate cancer. This consistency across studies not only reinforces the validity of the current study’s outcomes but also highlights the recurring significance of a HOST-response as a potentially critical factor in the prognosis of prostate cancer. These consistent observations suggest that further investigation into the biological mechanisms underlying a HOST-response could provide valuable insights into its role in cancer progression and treatment outcomes.

The established standard for managing prostate cancer patients after radical prostatectomy involves administering salvage radiation therapy when PSA levels escalate to 0.1 ng/mL [31,32,33]. This approach is favored by a meta-analysis prioritizing the PFS as its main outcome [34,35,36]. However, insights from a recent comprehensive study spanning multiple nations and institutions have prompted a reevaluation of this standard. This study demonstrated that adjuvant radiation therapy, when compared to early salvage radiation therapy, substantially decreases the risk of all-cause mortality among patients exhibiting advanced disease markers, specifically those with a Gleason score between 8 and 10 and a tumor stage of pT3 or pT4 [37]. These findings were significant even after adjustments were made for known prognostic factors, suggesting a potential shift in treatment paradigms. Additionally, the NRG-GU008 trial is currently randomizing prostate cancer patients with pathologic node-positive disease following radical prostatectomy and a detectable PSA to receive treatment intensification with abiraterone/prednisone and apalutamide [38]. Given these recent developments, patients with a HOST-response might benefit from adjuvant radiation therapy or treatment intensification, as explored in the NRG-GU008 trial, and therefore should be considered for future randomized clinical trials. This approach not only aims to improve survival rates but also enhances the quality of life for patients with severe prognostic factors, marking a pivotal step forward in the clinical research of prostate cancer treatments.

## 5. Conclusions

The presence of a HOST-response in prostate cancer patients treated with radical prostatectomy was associated with reduced PFS. These results highlight the importance of identifying patients at risk of disease failure who may benefit from adjuvant treatment, warranting further investigation in a prospective randomized clinical trial.

## Figures and Tables

**Table 1:** Comparison of the distribution of baseline clinical and treatment characteristics stratified by the presence of histologically overt stromal response.

	With HOST-Response (n = 166)	Without HOST-Response (n = 182)	*p*
Age (years), median (IQR)	61 (56, 66)	61 (55, 67)	0.957
Baseline PSA, ng/mL, median (IQR)	8.15 (5.4, 13.5)	7.20 (4.7, 10.4)	0.010
Prostatectomy tumor stage, no. (%)			0.024
T2	52 (32%)	78 (44%)	
T3a or higher	112 (68%)	101 (56%)	
Prostatectomy Gleason score, no. (%)			0.026
7 or less	92(55%)	122 (67%)	
8–10	74 (45%)	60 (33%)	
Prostatectomy margin status, no. (%)			0.089
Negative	112 (72%)	107 (63%)	
Positive	44 (28%)	63 (37%)	
Prostatectomy nodal status, no. (%)			0.700
Negative (N0)	115 (79%)	117 (81%)	
Positive (N1)	31 (21%)	28 (19%)	
Prostatectomy tumor stage T3a or higher and Gleason score 8–10			0.008
Yes	43 (40%)	69 (58%)	
No	64 (60%)	50 (42%)	
Follow-up (month), median (IQR)	45.47 (24.13, 68.53)	32.63 (17.47, 60.50)	0.004

Abbreviations: HOST-response, histologically overt stromal response; PSA, prostate-specific antigen; IQR, interquartile range.

**Table 2:** Covariate-adjusted hazard ratios for progression-free survival using the Fine–Gray model.

Covariates	Univariable			Multivariable		
	HR	95% CI	*p*	SDHR	95% CI	*p*
Age (years)	1.013	0.9831–1.044	0.400	1.002	0.968–1.037	0.895
Baseline PSA, ng/mL						
<4	0.674	0.265–1.762	0.432	0.675	0.279–1.633	0.384
4–10	Reference	Reference		Reference	Reference	
>10	1.145	0.715–1.834	0.571	0.718	0.441–1.168	0.181
Prostatectomy tumor stage, no.						
T2	Reference	Reference		Reference	Reference	
T3a or higher	5.504	2.657–11.40	0.000	3.570	1.604–7.943	0.001
Prostatectomy Gleason score						
7 or less	Reference	Reference		Reference	Reference	
8–10	4.259	2.625–6.912	0.000	2.660	1.596–4.433	0.000
Prostatectomy margin status						
Negative	Reference	Reference		Reference	Reference	
Positive	1.525	0.967–2.401	0.068	1.041	0.636–1.705	0.871
HOST-response						
No	Reference	Reference		Reference	Reference	
Yes	2.787	1.704–4.557	0.000	2.099	1.260–3.495	0.004

Abbreviations: HR, hazard ratio; CI, confidence interval; SDHR, subdistribution hazard ratio; PSA, prostate-specific antigen; HOST-response, histologically overt stromal response.

## Data Availability

The original contributions presented in the study are included in the article. Further inquiries can be directed to the corresponding author.
